# The role of the pulmonary function laboratory in the management of cystic fibrosis

**DOI:** 10.36416/1806-3756/e20260101

**Published:** 2026-05-22

**Authors:** Rodrigo Abensur Athanazio

**Affiliations:** 1 Unidade de Doencas de Vias Aereas, Divisao de Pneumologia, Instituto do Coracao - InCor - Hospital das Clinicas, Faculdade de Medicina, Universidade de Sao Paulo - HCFMUSP - Sao Paulo (SP) Brasil.

## BACKGROUND

Cystic fibrosis (CF) is a multisystem, autosomal recessive disease caused by dysfunction of the cystic fibrosis transmembrane conductance regulator (CFTR) protein and leading to impaired mucociliary clearance, chronic airway infection, and progressive structural lung damage. Despite substantial improvements in survival because of early diagnosis, multidisciplinary care, and highly effective CFTR modulators, respiratory disease remains the primary determinant of long-term morbidity and mortality in patients with CF.^1^

Pulmonary function testing plays a central role in the longitudinal assessment of patients with CF, contributing to disease characterization, risk stratification, and therapeutic guidance. Traditionally, spirometry-particularly FEV_1_-has been the cornerstone of functional monitoring and remains a robust predictor of mortality and transplant-free survival. However, it is increasingly recognized that spirometric indices lack sensitivity for detecting early or mild lung disease, especially in patients with preserved FEV_1_, a phenotype that has become more prevalent in the current treatment era.^2^

In this evolving clinical context, the pulmonary function laboratory has expanded beyond spirometry to incorporate techniques capable of detecting subtle abnormalities in ventilation distribution and small airway involvement, allowing a more comprehensive physiological assessment of CF lung disease (Chart 1).^2^ ^,^ ^3^

**Chart 1:** Pulmonary function tests used in the assessment and monitoring of cystic fibrosis lung disease.

Pulmonary function test	Main physiological target	Typical findings in cystic fibrosis	Clinical utility	Added value according to disease stage
Spirometry (FEV_1_, FVC, FEV_1_/FVC)	Airflow limitation in large and intermediate airways	Normal or mildly reduced values in early disease; progressive obstructive pattern in advanced disease	Core tool for routine monitoring, prognostic assessment, and therapeutic decision-making	Essential at all stages; limited sensitivity in early or mild disease
Spirometry with bronchodilator	Reversible airflow obstruction	Usually absent or minimal bronchodilator response	Helps exclude asthma overlap and assess treatment response in selected cases	Adjunctive role; limited impact on CF-specific management
Lung volume measurement (plethysmography)	Air trapping and hyperinflation (RV, RV/TLC, TLC)	Increased RV and RV/TLC reflecting small airway obstruction; TLC usually preserved until advanced disease	Improves disease characterization beyond spirometry	Particularly useful in moderate to advanced disease
DL_CO_ measurement	Alveolar-capillary gas exchange	Usually preserved; may be reduced in advanced disease with parenchymal destruction	Helps assess disease severity and differential diagnoses	Limited role in early disease; supportive in advanced stages
LCI	Ventilation inhomogeneity, small airway dysfunction	Elevated values despite normal spirometry; sensitive to early structural disease	Early detection of lung involvement; monitoring disease progression and treatment response	High value in early and mild disease; complementary at all stages
Exercise tests (6MWT, shuttle tests)	Integrated cardiopulmonary and systemic response to exercise	Often normal in mild disease; reduced performance in advanced stages	Prognostic information; correlates with quality of life and functional capacity	More informative in moderate to advanced disease
Serial longitudinal assessment	Rate of functional change over time	Decline in FEV_1_ or worsening LCI predicts disease progression	Risk stratification and long-term follow-up	Crucial across all disease stages

6MWT: six-minute walk test; LCI: lung clearance index; and CF: cystic fibrosis.

## OVERVIEW

A 19-year-old man with CF, homozygous for the F508del mutation and diagnosed with CF through newborn screening, was referred for routine follow-up. He reported mild daily cough without sputum production, no exacerbations in the previous year, and regular physical activity. He was receiving CFTR modulator therapy and had a normal BMI. 

Spirometry revealed preserved lung function, with an FEV_1_ of 96% of the predicted value and a normal FEV_1_/FVC ratio, with no significant bronchodilator response. Lung volumes as assessed by plethysmography were within normal limits, and DL_CO_ was preserved. Despite these reassuring findings, multiple-breath washout testing showed an elevated lung clearance index (LCI), consistent with ventilation inhomogeneity and early small airway dysfunction. 

This case illustrates a common and increasingly encountered scenario in adult CF care: patients with minimal symptoms and normal spirometry who nonetheless exhibit physiological evidence of early lung disease. The LCI has emerged as a sensitive and reproducible marker of ventilation heterogeneity, capable of detecting functional impairment before spirometric decline. In CF patients, elevated LCI values have been associated with structural abnormalities on chest CT scans, increased risk of disease progression, and response to therapeutic interventions, particularly in those with mild disease.^3^ ^,^ ^4^

In pediatric patients with CF, the LCI is particularly valuable for monitoring early lung disease because it detects subtle ventilation abnormalities before spirometric impairment becomes evident, thereby facilitating earlier and more targeted therapeutic interventions.^5^

## CLINICAL MESSAGE

The pulmonary function laboratory is a cornerstone of comprehensive CF care, extending well beyond conventional spirometry. While FEV_1_ remains essential for prognostic assessment and long-term monitoring, it is insufficient to fully characterize early or mild CF lung disease.^1^ ^,^ ^2^

A multimodal functional approach-including spirometry, lung volume measurement, DL_CO_ measurement, and selected use of multiple-breath washout testing and derived LCI-provides a more complete understanding of disease physiology, particularly in patients with preserved lung function. Such an approach supports earlier detection of small airway involvement, informs clinical decision-making, and enhances the evaluation of treatment response in clinical practice and research settings.^1^ ^-^ ^3^
